# The Limits of Reductionism in Medicine: Could Systems Biology Offer an Alternative?

**DOI:** 10.1371/journal.pmed.0030208

**Published:** 2006-05-23

**Authors:** Andrew C Ahn, Muneesh Tewari, Chi-Sang Poon, Russell S Phillips

## Abstract

In the first of a two part series, Ahn and colleagues discuss the reductionist approach pervading medicine and explain how a systems approach (as advocated by systems biology) may complement reductionism.

*This is the first in a series of two articles that look at the lessons for clinical medicine from systems biology.*

Since Descartes and the Renaissance, science, including medicine, has taken a distinct path in its analytical evaluation of the natural world [ 1, 2]. This approach can be described as one of “divide and conquer,” and it is rooted in the assumption that complex problems are solvable by dividing them into smaller, simpler, and thus more tractable units. Because the processes are “reduced” into more basic units, this approach has been termed “reductionism” and has been the predominant paradigm of science over the past two centuries. Reductionism pervades the medical sciences and affects the way we diagnose, treat, and prevent diseases. While it has been responsible for tremendous successes in modern medicine, there are limits to reductionism, and an alternative explanation must be sought to complement it.

The alternative explanation that has received much recent attention, due to systems biology, is the systems perspective ( Table 1). Rather than dividing a complex problem into its component parts, the systems perspective appreciates the holistic and composite characteristics of a problem and evaluates the problem with the use of computational and mathematical tools. The systems perspective is rooted in the assumption that the forest cannot be explained by studying the trees individually.

**Table 1:**
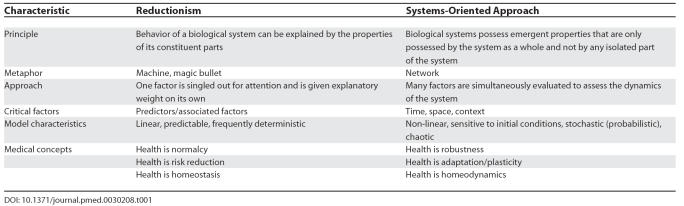
Reductionism versus a Systems-Oriented Perspective

In order for a systems perspective to be fully appreciated, however, we must first recognize the reductionist nature of medical science and understand its limitations. For this reason, the first article in this series is dedicated to examining the reductionist approach that pervades medicine and to explaining how a systems approach (as advocated by systems biology) may complement it. In the second article, we aim to provide a more practical discussion of how a systems approach would affect clinical medicine. We hope that these discussions can stimulate further inquiry into the clinical implications of systems principles.

## Current Medical Science

While the *implementation* of clinical medicine is systems-oriented, the science of clinical medicine is fundamentally reductionist. This is shown in four prominent practices in medicine: (1) the focus on a singular, dominant factor, (2) emphasis on homeostasis, (3) inexact risk modification, and (4) additive treatments.

### Focus on a singular factor

When the human body is viewed as a collection of components, the natural inclination of medicine is to isolate the single factor that is most responsible for the observed behavior. Much like a mechanic who repairs a broken car by locating the defective part, physicians typically treat disease by identifying that isolatable abnormality. Implicit within this practice is the deeply rooted belief that each disease has a potential singular target for medical treatment. For infection, the target is the pathogen; for cancer, it is the tumor; and for gastrointestinal bleeding, it is the bleeding vessel or ulcer.

While the success of this approach is undeniable, it leaves little room for contextual information. A young immuno-compromised man with pneumococcal pneumonia usually gets the same antibiotic treatment as an elderly woman with the same infection. The disease, and not the person affected by it, becomes the central focus. Our contemporary analytical tools are simply not designed to address more complex questions, and, thus, questions such as “how do a person's sleeping habits, diet, living condition, comorbidities, and stress collectively contribute to his/her heart disease?” remain largely unanswered.

### Emphasis on homeostasis

For decades, homeostasis has been a vital, guiding principle for medicine. Claude Bernard in 1865 and later Walter B. Cannon popularized this principle, expounding on the body's remarkable ability to maintain stability and constancy in the face of stress [ 3]. Since then, homeostasis has been incorporated into clinical practice. Illness is defined as a failed homeostatic mechanism, and treatment requires physicians to substitute for this failed mechanism by correcting deviations and placing parameters within normal range. This corrective treatment approach is true for a range of medical conditions, from hypothyroidism to hypokalemia to diabetes.

This interpretation of homeostasis, however, is biased by a reductionist viewpoint in two ways. First, the emphasis on correcting the deviated parameter (e.g., low potassium) belies the importance of systemswide operations. Either alternate, less intuitive targets may be more effective, or correction of the deviated parameter may itself have harmful system-wide effects. Existing evidence that demonstrates adverse effects of calcium for hypocalcemia [ 4, 5] or blood pressure control for strokerelated hypertension [ 6] points to the limitations of this homeostasis interpretation as a universal principle.

Secondly, the exclusive focus on normal ranges belies the importance of dynamic stability. Because reductionism often disregards the dynamic interactions between parts, the system is often depicted as a collection of static components. Consequently, emphasis is placed on static stability/normal ranges and not on dynamic stable states, such as oscillatory or chaotic (seemingly random but deterministic) behavior. Circadian rhythms [ 7] are an example of oscillatory behavior, and complex heart rate variability [ 8–10] is an example of chaotic behavior. Failure to include these dynamic states in the homeostasis model may lead to treatments that are either ineffective or even detrimental.

### Inexact risk modification

Since disease cannot always be predicted with certainty, health professionals must identify and modify risk factors. The common, unidimensional, “one-riskfactor to one-disease” approach used in medical epidemiology, however, has certain limitations.

An example is hypertension, a known risk factor for coronary heart disease. Guidelines suggest pharmacological and lifestyle treatment for individuals with systolic blood pressure greater than 140. This strategy is supported by evidence from the Framingham Study, which showed that men between 35 and 64 years of age with systolic blood pressures greater than 140 were twice as likely to develop heart disease as compared to individuals with systolic blood pressure less than 140 [ 11]. However, given that nearly 70% of the American population is not affected by hypertension, up to 30% of coronary artery disease develops in individuals with normal blood pressure [ 11]. Conceivably, a large number of people at small risk may give rise to more cases of disease than a small number of people at high risk. This observation is termed the prevention paradox [ 12].

To capture these missed cardiac events, the natural recourse is to progressively lower the blood pressure threshold for treatment. Consequently, the Joint National Committee on Prevention, Detection, Evaluation, and Treatment of High Blood Pressure lowered its initial diastolic blood pressure threshold of 105 in 1977 to 90 in 1980, to 85 (for high normal) in 1992, and to 80 (for prehypertension) in 2003. The cost of such a strategy is the unnecessary treatment of individuals who wouldn't have developed coronary disease in the first place. This problem originates from the constraints imposed by a one-risk to one-disease analysis and the inability to work with multiple risk factors and calculate their collective influences. If a more multidimensional analytical method were used, then more precise risk projections for individuals could be devised.

### Additive treatments

In reductionism, multiple problems in a system are typically tackled piecemeal. Each problem is partitioned and addressed individually. In coronary artery disease, for example, each known risk factor is addressed individually, whether it be hyperlipidemia or hypertension. The strategy is also extended to coexisting diseases, such as hypothyroidism, diabetes, and coronary artery disease. Each disease is treated individually, as if the treatment of one disorder (such as coronary artery disease) has minimal effects on the treatment of another (such as hypothyroidism). While this approach is easily executable in clinical practice, it neglects the complex interplay between disease and treatment. The assumption is that the results of treatments are additive rather than nonlinear.

## Limitations to Current Medical Science

The science underlying our medical practices, from diagnosis to treatment to prevention, is based on the assumption that information about individual parts is sufficient to explain the whole. But there are circumstances in which the complex interplay between parts yields a behavior that cannot be predicted by the investigation of the parts alone. The failure to account for these circumstances is the common denominator for the explanations of why the aforementioned practices are, in many cases, inadequate.

So how should these complexities be addressed? Is there a formal method that can explain how the pieces create the whole? How do we shift our lens from the parts to the system? The answers to these questions may come from a relatively new branch of science called systems biology [ 13–16]. Systems biology was conceived to address the molecular complexities seen in biological systems. One major impetus for its creation was the human genome project.

## Human Genome Project

The completion of the human genome project in 2003, in addition to the development of high-throughput technologies such as DNA array chips, has led scientists to confront a challenge they could not address before; namely, how do genes interact to collectively create a system-wide behavior?

The human genome contains 30,000 to 35,000 genes [ 17]. Although this number is just five times the number of genes in a unicellular eukaryote (e.g., approximately 6,000 genes in Saccharomyces cerevisiae) [ 18], the human genome encodes for nearly 100 trillion cells in the human body [ 19]. The richness of information is derived not only in the genes themselves but also in the interaction between genes and between their respective products. The genes encode for messenger RNA, the messenger RNAs encode for proteins, and the proteins act as catalysts or secondary messengers, among other diverse functions. Between each hierarchical level, modifications (e.g., alternative splicing) are made, and at each hierarchical level (e.g., transcription), thousands of molecules interact with other molecules to create a complex regulatory network. What becomes evident from these molecular analyses is that phenotypic traits emerge from the collective action of multiple individual molecules [ 20]. Therefore, the previous notion that a single genetic mutation is responsible for most phenotypic defects is overly simplistic. Complex diseases such as cancer, asthma, or atherosclerosis cannot generally be explained by a single genetic mutation.

## Systems Biology: An Introduction

The need to make sense of complex genetic interactions has led some researchers to shift from a componentlevel to system-level perspective. This novel approach incorporates the technical knowledge obtained from systems engineering, which began with Norbert Weiner's “cybernetics” in 1948 and Ludwig von Bertalanffy's “General Systems Theory” in 1969 [ 21, 22]. The developing fields of chaos theory, nonlinear dynamics, and complex systems science, along with computational science, mathematics, and physics, have also contributed to the analytical armamentarium used by systems analysts.

The intention of applying these theories to biological systems (termed “systems biology”) is to understand how properties emerge from the nonlinear interaction of multiple components ( Table 2). How does consciousness arise from the interactions between neurons? How do normal cellular functions such as cellular division, cell activation, differentiation, and apoptosis emerge from the interaction of genes? These questions highlight the difficulty of understanding complex biological systems—the moment the lens is directed toward the components of a biological system, the behaviors and properties of the whole system become obscure. Plainly said, one loses sight of the forest for the trees.

**Table 2:**
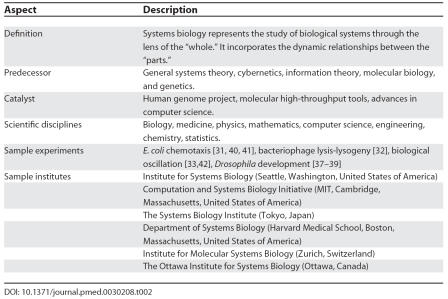
Overview of Systems Biology

Systems biology is an integrative approach that combines theoretical modeling and direct experimentation. Theoretical models provide insights into experimental observations, and experiments can provide data needed for model creation or can confirm or refute model findings. With this integrative approach, it becomes apparent that no single discipline is ideal to address systems biology. Scientists from molecular biology, computational science, engineering, physics, statistics, chemistry, and mathematics need to cooperate in order to explain how the biological whole materializes [ 23].

While the field of systems biology is young, it has been received with substantial enthusiasm. Many believe that, without a system-level understanding, the benefits of the genomic information cannot be fully realized. The perceived importance of this understanding is reflected in the investments made by major academic and industrial centers within the past few years [ 24].

## Importance of Context, Space, and Time

How is systems-level understanding achieved? The answer likely lies in the dynamic and changing nature of biological networks. Unlike the static depiction of many wiring network representations, both the molecular concentrations and enzyme activities are continually changing as a result of influences from other molecular substrates. The network is an interactive and dynamic web in which the properties of a single molecule are contingent on its relationship to other molecules and the activities of those other molecules within the network. Therefore, the behavior of the system arises from the active interactions of these biological components. To elicit the system-wide behavior, three factors need to be considered: (1) context, which values the inclusion of all components partaking in a process; (2) time, which considers the changing characteristics of each component; and (3) space, which accounts for the topographic relationships between and among components. Box 1 and Figure 1 show an example of how systems methods—incorporating context, time, and space—allowed researchers to provide a mechanistic explanation for Escherichia coli chemotaxis.

**Figure 1:**
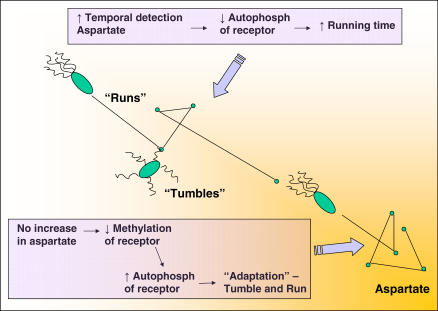
E. coli Chemotaxis E. coli has been observed to migrate toward areas of higher aspartate concentrations through a series of “runs” and “tumbles” (see Box 1). Autophosph, autophosphorylation.

The three factors of context, time, and space play a vital role in systems science. Systems biologists consequently use tools such as differential equations, diffusion functions, computational models, and high throughput tools to incorporate one or more of these factors to address a research question. This approach differs from traditional medical methods, where the central focus is elaborating the instantaneous property of a component involved in a disease process. In many medical models, the process of data extraction, such as obtaining serum glucose level or blood pressure, can lead to loss of information on time, space, or context. Systems biologists contend that loss of this information leads to loss of rich information that would otherwise contribute to a better understanding of the systemic and dynamic behavior of the human body.

## Systems Biology Concepts

Several concepts have emerged in systems biology to describe properties occurring at the systems level. One prominent concept is robustness, defined as the ability to maintain stable functioning despite various perturbations [ 25, 26]. Natural systems specifically demonstrate an uncanny penchant for robustness, which, as many have argued, is necessary for natural systems to survive and procreate [ 27]. Robustness is attained by five described mechanisms: feedback control, structural stability, redundancy, modularity, and adaptation (see Box 2) [ 13, 28]. Biological systems across all scales, from cells to organisms, rely on a combination of these mechanisms to maintain a semblance of stability. The human body is no exception.

The stability discussed in systems biology is distinct from the stability commonly perceived in clinical medicine. Medical practitioners often picture stability as an unwavering entity such that values are maintained within a specific, confined range. But stability in systems biology is revealed dynamically, and it is the *behavior* of the system rather than the *state* of the system that remains consistent. This dynamic stability can assume many forms, including homeostatic, bistable (having two stable states), oscillatory, or chaotic [ 29]. Normal biological functions can be classified into one of these dynamic behaviors: for instance, bacteriophage lysis-lysogeny as bistable, circadian rhythms as oscillatory, or heart rate variability as chaotic. This varied perspective of stability is more extensive than the commonly accepted notion of homeostasis and may ultimately influence how treatments are deliberated.

## Lessons from Systems Biology

The fundamental disconnect that exists between clinical medicine and systems biology largely stems from their disparate worldviews—one focuses on the parts and the other on the systems. As a consequence, the factors of time, space, and context, which are considered vital for a system-level understanding, are not assigned the same level of importance in medicine as they are in systems biology. Moreover, system-level concepts such as robustness, stability, and variability do not have meaningful equivalents in the medical vernacular. The incorporation of such concepts into medicine may help address certain limitations and greatly enhance its therapeutic potential. The second article in this series will explore how systems medicine may be realized in practice.

**Box 1. Chemotaxis as an Example of Systems Biology's Application**

E. coli chemotaxis is an example of systems biology's application (see Figure 1). Chemotaxis is defined as directed motion of a cell toward increasing (or decreasing) concentrations of a particular chemical substance. E. coli has been observed to migrate toward areas of higher aspartate concentrations through a series of “runs” and “tumbles.” The “runs” are linear paths taken by the bacteria, while the “tumbles” are random rotations that reorient the bacteria. When bacteria reach higher concentrations of aspartate, time spent “running” in proportion to “tumbling” increases—the logic being that if higher concentrations of aspartate are encountered, the bacterium is on the right track and should continue in that direction. If the E. coli fails to detect increasing aspartate concentrations, the bacterium eventually exhibits “adaptation,” where it returns to the baseline “tumble and run” activities. This ensures that it does not continually head in the wrong direction.

Conventional medical methods have, for more than a decade, been able to identify the enzymes and molecules involved in the chemotactic pathway. Despite this, little was known about how the interactions in this pathway translated to its known chemotactic behavior, namely the ability of E. coli to “adapt” in a large range of aspartate concentrations. Spiro, et al. [ 31] used systems methods in 1997 to provide a mechanistic explanation. They placed the involved enzymes into a mathematical equation (context), considered the relationship between these enzymes (space), and analyzed the activities for each enzyme with the use of computational tools (time). Increased temporal detections of aspartate led to reduced autophosphorylation rate of the aspartate receptor. This effect reduced the tumbling rate and increased the running time. When there was no increased detection of aspartate, methylation of the aspartate receptor occurred, which increased the autophosphorylation rate and caused the E. coli to return to prestimulus tumble-and-run activities (adaptation). Importantly, this adaptive behavior occurred at different aspartate concentrations, explaining how E. coli does not perpetually exist in an excited state, even at higher aspartate concentrations.

Similar conceptual breakthroughs have been obtained with the use of systems methods in other biological phenomena, such as bacteriophage lysis-lysogeny [ 32], biological oscillations [ 33, 34], circadian rhythms [ 35, 36], and Drosophila development [ 37–39]. In these situations, the incorporation of context, time, and space into the equation has provided information not otherwise obtained through structural information alone.

**Box 2**

**Feedback control:** Serves to correct deviations and restores the system to its natural behavior.

**Structural stability:** Explains for the stability that arises from the very nature of the network structure. For instance, the World Wide Web was shown to be resistant to random attacks to Web sites by virtue of its organization [ 30].

**Redundancy:** Allows for functionally equivalent units to substitute for one another in the event of a failure.

**Modularity:** Prevents amplification of a perturbation by dividing function or structure into subunits or modules.

**Adaptation:** Promotes survival and functioning in a variety of environmental conditions.

## Abbreviation

MIT: Massachusetts Institute of Technology
